# Supplementary material for: Ten simple rules for computational biologists collaborating with wet lab researchers
Source: PLoS Comput Biol. 2024 Jun 20;20(6):e1012174. doi: 10.1371/journal.pcbi.1012174 (PMC11189214; doi:10.1371/journal.pcbi.1012174)
Supplement: S1 Table — The idea here is that there are no good answers (well, sometimes there are), but that each party fills the form and then compares their answer, to hopefully align expectations. Some questions are phrased rhetorically, but meant to explicitly prepare all members for a collaboration’s potential tension points. To fill put an “x” in the appropriate column, where 3 is “agree equally with both statements,” 1 is “strongly agree with statement on left,” and 5 is “strongly agree with statement on right.” (PDF) [file pcbi.1012174.s001.pdf]

Supplementary Table 1: Collaboration expectations form. The idea here is that there are no good answers (well, sometimes there are), but that each party fills the form and then compares their answer, to hopefully align expectations. Some questions are phrased rhetorically, but meant to explicitly prepare all members for a collaboration's potential tension points. To fill put an 'x' in the appropriate column, where 3 is 'agree equally with both statements', 1 is 'strongly agree with statement on left', and 5 is 'strongly agree with statement on right'.

	1	2	3	4	5	
**Choosing collaborators**						
The questions on this form must be adhered to as agreed						Questions on this form are only for orientation
Scope and whether to continue collaborating should be discussed regularly						Once the collaboration has started, it will be seen through to a deliverable, no matter what happens
**Experimental design**						
The wet lab people determine the experimental design/strategy, the dry lab people determine the analysis strategy						Both experimental and analysis strategies are to be discussed and jointly agreed on by both parties
Analysis plan and experimental design are to be discussed once the data have been collected						Analysis plan and experimental design should be discussed and fixed prior to data collection
Analysts are blinded whenever possible						All metadata is always visible to analysts
**Communication and functioning**						
Meetings should be held on a regular (e.g. monthly/biweekly) basis, and simply be brief if there was no development						Meetings should be organized whenever they are needed (e.g. to discuss results or problems)
Both parties should always adhere to agreed deadlines						Agreed deadlines are guidelines rather than absolute targets
To each their expertise						All parties are expected to try to learn and understand what the others are doing
As much as possible, every piece of data/analysis should be made available to everyone						Results are to be exchanged/communicated between partners in the form of publication-ready figures and descriptions
Emails are expected to be answered on the same day						Emails are expected to be answered within a week or two
Both parties agree on tools to track progress (i.e. Trello) and collaborative writing (i.e. Google Docs, Overleaf)						Each team uses their preferred software
Predefined file naming conventions						No convention but file names are clear
Metadata delivered in machine readable format						Metadata sent as is collected
Manuscript written in a collaborative document allowing concurrent editing						Different versions of manuscripts are individual documents shared by email
**Scientific values and publication strategy**						
What matters is excellent science - and hence high impact factor						What matters is excellent science - and hence sound, robust results
We should make code/data available if we are forced to						By default everything should be openly available, latest by publication time (whether pre-print or final paper)
Everything should be published as pre-print						Never mention anything unless it's actually published by a journal
The people doing the experiments are first authors, the dry lab people are somewhere after that						The people analyzing and making sense of the data are first authors or first co-authors
Negative or inconclusive results are sadly unglamorous, but happen and are part of science						If you have negative or inconclusive results, it means you haven't looked hard enough or given it proper attention
**Intellectual contribution and attribution**						
Projects are owned and conceived by the wet lab						Projects are owned and conceived by the dry lab
Results are interpreted by the dry lab						Results are interpreted by the wet lab
Figure making is a graphic design task (using an image editor)						Figure making is a data analysis task (using ggplot, GraphPad, etc)
**Etiquette**						
Direct communication is not harsh, just direct						Well thought, polite phrasing is expected in all communication exchanges
Excluding collaboration members from some exchanges (meetings, messages) is ok						All exchanges should be public and no member can be excluded
Freedom to give feedback regardless of career stage						Mind status when giving feedback
